# Pleomorphic Appearance of Breast Cancer Cutaneous Metastases

**DOI:** 10.7759/cureus.20301

**Published:** 2021-12-09

**Authors:** Philip R Cohen

**Affiliations:** 1 Dermatology, University of California, Davis Medical Center, Sacramento, USA

**Keywords:** zosteriform, telangiectoides, skin, metastases, erysipelatoides, en cuirasse, cutaneous, carcinoma, alopecia, breast cancer

## Abstract

Cutaneous metastases occur in approximately 10% of oncology patients as a feature of a persistent solid tumor or the harbinger of recurrent neoplastic disease. However, they can be the presenting manifestation of an unsuspected visceral malignancy in one percent of previously cancer-free individuals. Metastatic skin lesions from breast carcinoma are diverse in their appearance. The clinical presentation of cutaneous metastases in three women with breast cancer is described and both the morphology of skin metastases caused by breast carcinoma and the conditions that are mimicked by breast cancer cutaneous metastases are reviewed. Skin metastases from breast carcinoma commonly appear as firm, flesh-colored to red, smooth or ulcerated or crusted, nodules, papules, and plaques on the ipsilateral chest wall and breast. However, unique sites of breast cancer cutaneous metastases are the eyelids, inframammary folds, ipsilateral lymphedematous arm, scalp, subungual nail bed, and umbilicus; in addition, skin metastases can occur in mastectomy scars and radiation therapy ports. Carcinoma erysipelatoides, carcinoma telangiectoides, and carcinoma en cuirasse are classic patterns of skin metastases that can be observed in breast cancer patients; carcinoma hemorrhagiectoides is a recently observed skin metastases pattern that has also been noted in oncology patients with breast carcinoma. The pleomorphic skin lesions of breast cancer metastases can masquerade as benign cutaneous lesions and tumors (such as a collision tumor, cyst, dermatofibroma, and milia-en-plaque), cutaneous malignancies (such as melanoma and non-melanoma skin cancers), infections (such as cellulitis, folliculitis, herpes zoster, and paronychia), reactive erythema (such as erythema annulare centrifugum, and urticaria), skin conditions (such as alopecia areata, dermatitis, hidradenitis suppurativa, and scleroderma), and vascular lesions (such as angiokeratoma, angiosarcoma, lymphangioma circumscriptum, purpura, and pyogenic granuloma). In addition, breast carcinoma cutaneous metastases can not only mimic other miscellaneous conditions such as erosions and ulcers, Paget’s disease, and papillomatosis cutis lymphostatica but also have unusual morphology such as targetoid lesions or a sharply demarcated red infiltration of the nasal tip similar to a clown’s nose. The possibility of a breast cancer cutaneous metastasis should be considered in the evaluation of a patient with breast cancer--and although less likely, in a cancer-free individual--who develops a new and/or a treatment-unresponsive cutaneous lesion. A biopsy of the skin lesion is necessary to confirm the diagnosis of breast cancer cutaneous metastasis.

## Introduction

Cutaneous metastases from visceral malignancy are uncommon. They can be the clinical presentation of progressive neoplasm or the harbinger of recurrent disease in an oncology patient. Rarely they are the presenting manifestation of a previously undiagnosed cancer [1-3].

Breast cancer is the most frequent malignancy in women. It is also a common neoplasm associated with cutaneous metastases. The morphology of the skin lesions secondary to metastatic breast cancer is variable in appearance [1-20].

Cutaneous metastases that developed in three women with breast cancer are described. They appeared as annular erythema, carcinoma en cuirasse, carcinoma erysipelatoides, an erythematous targetoid lesion, and/or zosteriform metastases. The pleomorphic appearance of breast cancer cutaneous metastases is reviewed.

## Case presentation

Case 1

A 47-year-old Hispanic woman presented for evaluation of possible cellulitis involving her left breast. She had been diagnosed with invasive ductal breast cancer of her left breast 10 months earlier. Her tumor was positive for estrogen receptor and negative for both progesterone receptor and human epidermal growth factor receptor 2 (HER2).

Since her diagnosis, she was initially treated with weekly paclitaxel for 12 weeks. She then received every three weeks four cycles of 5-fluorouracil, epirubicin, and cyclophosphamide (FEC). She was scheduled for a mastectomy in six weeks; however, a month after the final treatment she developed an asymptomatic red rash on her left breast.

**Figure 1:**
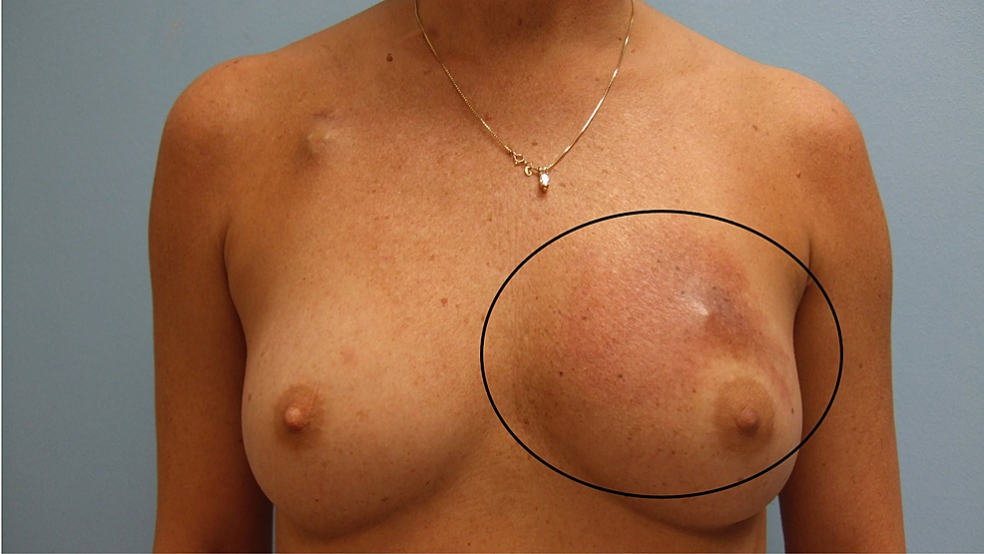
Breast cancer cutaneous metastasis presenting as carcinoma erysipelatoides A frontal view of a non-tender red patch on the superior aspect of the left breast (black oval) of a 47-year-old Hispanic woman who had been diagnosed with invasive ductal breast cancer of her left breast 10 months earlier and had recently completed chemotherapy prior to a planned mastectomy. Her oncologist suspected cellulitis, and she was sent for additional evaluation.

**Figure 2:**
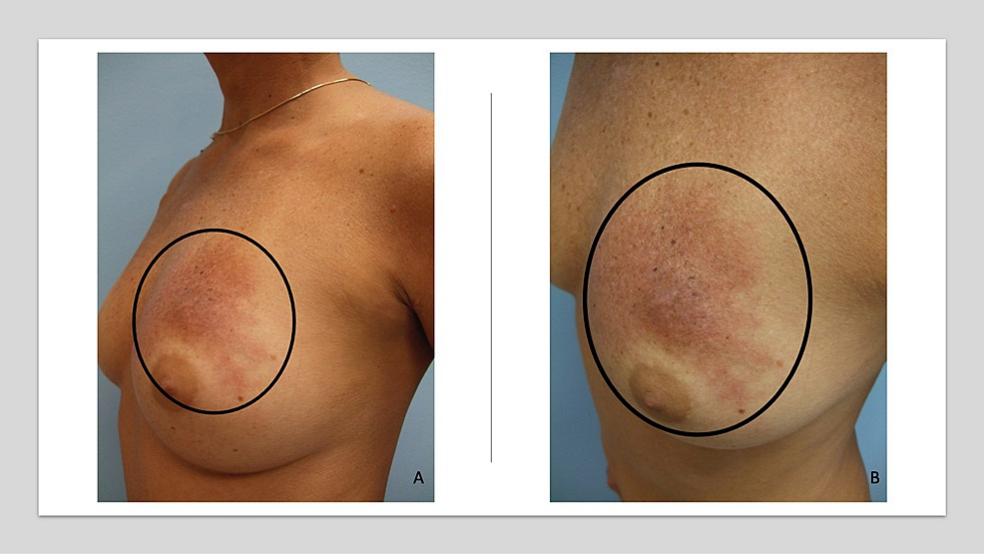
Carcinoma erysipelatoides masquerading as an erysipelas infection of the left breast in a woman with invasive ductal breast cancer Distant (A) and closer (B) lateral view of the left breast in a woman with invasive ductal left breast carcinoma shows a painless erythematous patch above the areola (black oval) that appeared the following 12 weeks of paclitaxel and four cycles of 5-fluorouracil, epirubicin, and cyclophosphamide (FEC). The skin biopsy showed metastatic cancer cells--consistent with breast origin--in the dermal lymphatics. Her scheduled mastectomy was postponed, and she was treated with additional chemotherapy.

Cutaneous examination showed a painless erythematous patch on the superior aspect of her left breast above the areola (Figures 1, 2). The area was not warm. However, her oncologist suspected an infection and referred her to see a dermatologist.

A biopsy, using the punch technique, demonstrated metastatic cancer cells--consistent with breast origin--in the dermal lymphatics. Correlation of the clinical presentation and pathologic findings established the diagnosis of carcinoma erysipelatoides. The mastectomy was postponed, and her progressive breast carcinoma with new cutaneous breast cancer metastases was treated with additional chemotherapy.

Case 2

A 54-year-old Greek woman presented with extensive and progressively enlarging skin lesions on her chest and abdomen. Two years earlier, she had been diagnosed with triple-negative invasive ductal carcinoma of the left breast. Her tumor did not express estrogen receptor, progesterone receptor, or HER2.

She presented with her initial cutaneous breast cancer metastasis a year after diagnosis. This appeared as a nodule on her left chest wall. During the subsequent 12 months, she continued to develop new skin metastases.

**Figure 3:**
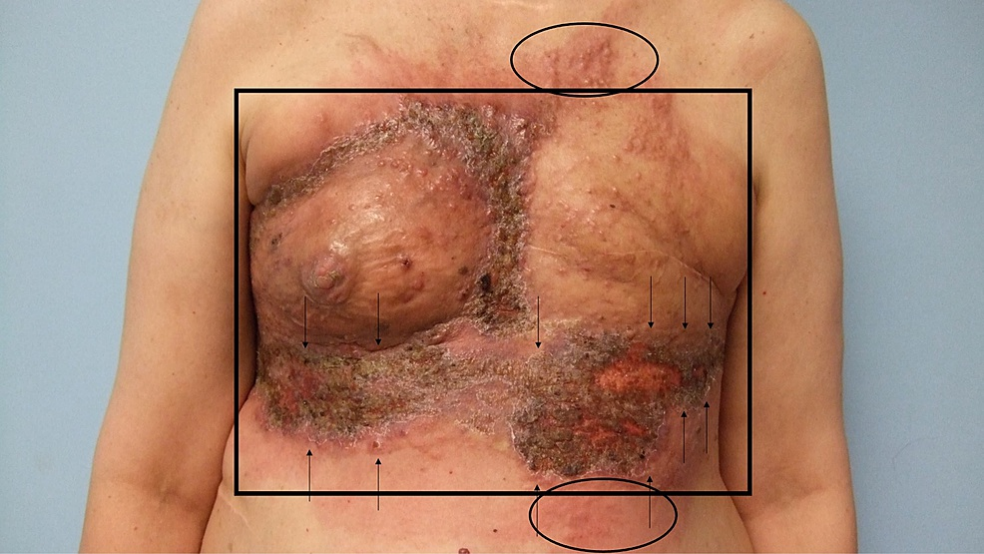
Breast cancer cutaneous metastasis presenting as carcinoma en cuirasse, carcinoma erysipelatoides, and zosteriform cutaneous metastases A frontal view of extensive and progressive cutaneous breast cancer metastases on the chest and upper abdomen, and bilaterally extending to the flanks of a 54-year-old Greek woman who had been diagnosed with triple-negative invasive ductal carcinoma of the left breast two years earlier and began to develop skin metastases one year later. Sclerodermoid metastases of carcinoma en cuirasse (within the black rectangle), erythematous cellulitis-like carcinoma erysipelatoides (within the black oval), and herpes zoster-like zosteriform cutaneous metastases (between the black arrows) are present.

**Figure 4:**
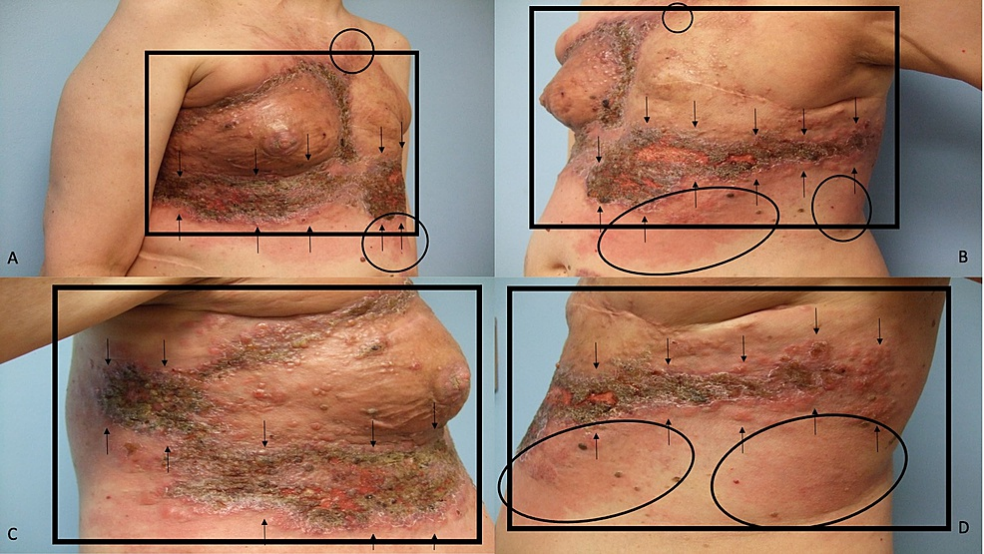
Carcinoma en cuirasse mimicking scleroderma, carcinoma erysipelatoides mimicking erysipelas, and zosteriform cutaneous metastases mimicking varicella-zoster virus infection on the chest, abdomen, and flanks of a woman with invasive ductal carcinoma of the left breast Distant (A and B) and closer (C and D) lateral views of the right side (A and C) and left side (B and D) of the chest, abdomen, and flank of a woman with triple-negative invasive left breast ductal carcinoma who has developed multiple patterns of breast cancer cutaneous metastases. The skin overlying her entire chest is firm, indurated, and bound down (black rectangle); these are the sclerodermoid changes of carcinoma en cuirasse. Erythematous macular patches (black ovals) mimicking erysipelas are present in the superior and inferior borders of her skin lesions; these are the cellulitis-like changes of carcinoma erysipelatoides. Eroded and crusted plaques with some pseudo-vesicular papules and nodules, occupying multiple consecutive dermatomes (between the black arrows), extend bilaterally from the mid-abdomen onto the lateral flanks; these are the herpes zoster-like changes of zosteriform cutaneous metastases. Skin biopsies showed metastatic tumor cells (consistent with breast carcinoma) between dermal collagen bundles and filling lymph vessels in the dermis.

Her metastatic skin lesions had several morphologies (Figures 3, 4). Her entire chest was firm and indurated, and the overlying skin was bound down. The superior and inferior borders of the skin lesions were erythematous and macular. Bilaterally, extending from the midline of her abdomen onto her lateral flanks were eroded and crusted plaques that occupied multiple consecutive dermatomes; some of the papules and nodules had a pseudo-vesicular appearance.

Punch biopsies of the affected areas demonstrated metastatic tumor cells (consistent with breast carcinoma) not only between the collagen bundles in the dermis but also filling the dermal lymphatics. Correlation of the clinical presentation and pathologic findings established the diagnoses of carcinoma en cuirasse, carcinoma erysipelatoides, and zosteriform cutaneous metastases.

Case 3

A 54-year-old Caucasian woman presented for evaluation of new skin lesions on her right breast and left chest. She had been in an automobile accident, in which the airbags from the car had contacted the affected areas, four months earlier. The itchy red rash had appeared three months later, and she thought the new lesions were dermatitis resulting from contact with the airbags during the car accident.

She had been diagnosed with invasive ductal breast cancer of her left breast two and a half years earlier. Management (during the year following diagnosis) had included chemotherapy, mastectomy of the left breast, and radiation therapy to the left chest wall. Thereafter, she received trastuzumab for four months and was currently being treated with daily anastrozole for the last 15 months.

**Figure 5:**
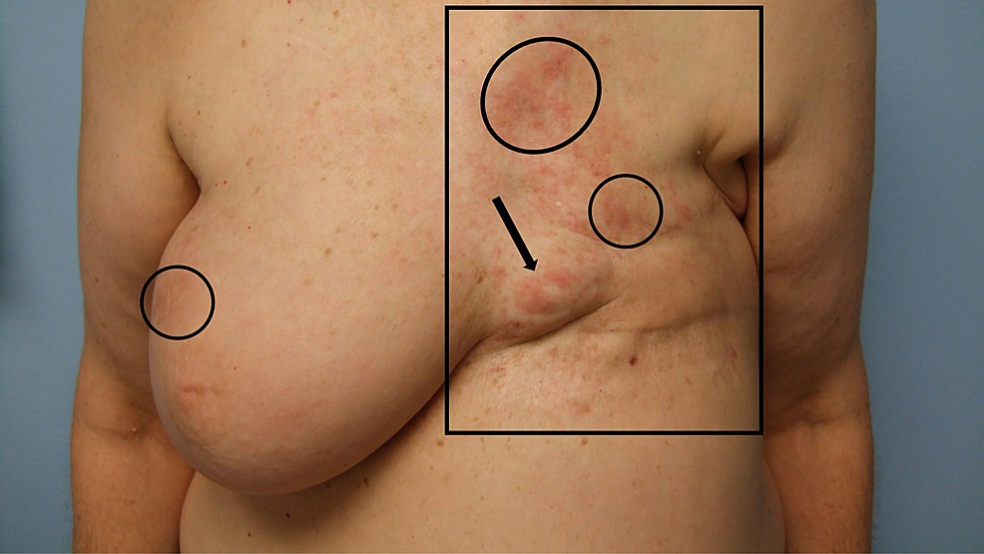
Breast cancer cutaneous metastasis presenting as targetoid and annular erythema in the radiation therapy port on the left chest and annular erythema on the right breast A frontal view of erythematous macular cutaneous breast cancer metastases, not only within the radiotherapy port (black rectangle) on the left chest, but also on the right breast of a 54-year-old Caucasian woman who had been diagnosed with invasive ductal left breast cancer two-and-a-half years ago and had been treated with preoperative and postoperative chemotherapy, left breast mastectomy, and radiation therapy to the left chest wall during the subsequent year; she currently had been receiving daily hormonal therapy for 15 months. The patient had experienced contact of her right breast and left chest with an airbag during an automobile accident four months earlier; the skin metastases appeared one month ago. Before establishing the diagnosis of breast cancer cutaneous metastases, the patient considered the pruritic erythematous annular (black ovals) and targetoid (black arrow) lesions to be dermatitis from contact with the airbag.

**Figure 6:**
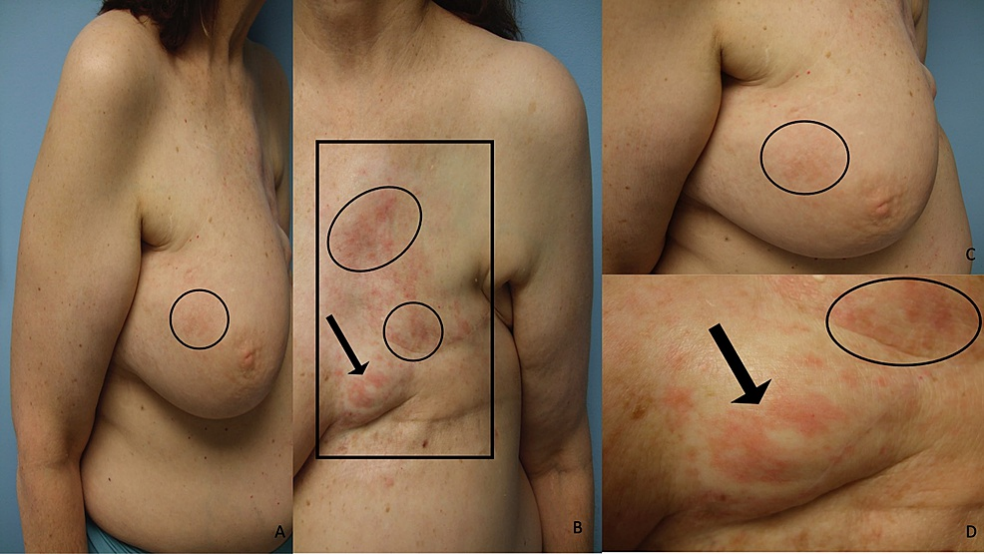
Radiotherapy port-associated annular and targetoid erythema on the left chest and annular erythema on the right breast are the presenting manifestations of cutaneous metastases in a woman with invasive ductal left breast carcinoma Distant (A and B) and closer (C and D) lateral view of the right breast (A and C) and frontal view of the left chest (B and D) of a woman with invasive ductal carcinoma of the left breast who has developed breast cancer cutaneous metastases not only within the radiation therapy port (black rectangle) on her left chest but also on her right breast. The red cutaneous metastases appeared as annular (black ovals) and targetoid (black arrow) macules which the patient initially considered to be dermatitis. The targetoid lesion was flat and appeared as a red ring surrounding the white central area. Right breast and left chest skin biopsies of the erythematous patches both showed metastatic carcinoma (compatible with the patient’s primary breast cancer) in the dermis and within the vascular spaces.

Cutaneous examination showed several erythematous patches on the right breast and the left chest (Figures 5, 6). Most of the new lesions on the left chest were within the boundaries of the radiation therapy port. One of the patches on the left chest was targetoid in appearance; the white center was surrounded by a red ring.

Biopsy of the erythematous patches on the right breast and the left chest both showed metastatic carcinoma (compatible with the patient’s primary breast cancer) in the dermis and within the vascular spaces. Correlation of the clinical presentation and pathologic findings established the diagnosis of cutaneous breast cancer metastases--both within and outside of the radiation therapy port--appearing as targetoid and annular erythema, which the patient initially interpreted to be dermatitis.

## Discussion

A retrospective study observed the incidence of cutaneous metastases to be 10% (420 of 4020) of oncology patients. In 7.6% (306 of 4020) of these individuals, the initial feature of extranodal metastatic cancer was skin metastasis. Indeed, in one percent (70 of 7316) of cancer patients, the cutaneous metastasis was the presenting manifestation of unsuspected cancer [1].

The morphology of metastatic skin lesions from breast carcinoma is diverse (Table 1) [1-20]. The most common presentation of breast cancer cutaneous metastases are nodules on the ipsilateral chest wall and breast; however, the skin metastases can also appear as papules or plaques. The lesions are firm and flesh-colored to red; occasionally, they become ulcerated or crusted [1-3].

**Table 1:** Breast cancer cutaneous metastases: mimicking conditions and skin manifestations CR: case report

Condition	References
Benign cutaneous lesions and tumors	[1-6]
Collision tumor	[3,4]
Cyst	[2]
Dermatofibroma	[5]
Milia-en-plaque	[6]
Nodule	[1-3]
Papule	[1-3]
Plaque	[1,2]
Cutaneous malignancy	[4,7,8]
Basal cell carcinoma	[7]
Keratoacanthoma	[8]
Melanoma	[7]
Squamous cell carcinoma	[4]
Infections	[1-3,9,10] CR
Carcinoma erysipelatoides	[2,3] CR
Folliculitis	[9]
Zosteriform metastases	[1,3,10] CR
Miscellaneous	[1-3,11-13] CR
Clown nose	[11]
Erosions	[2]
Paget’s disease	[3]
Papillomatosis cutis lymphostatica	[12]
Targetoid	[13] CR
Ulcers	[1]
Reactive erythema	[3,14,15] CR
Annular erythema	[3] CR
Erythema annulare centrifugum	[3,14]
Urticaria	[15]
Skin conditions	[1-4,16,17] CR
Alopecia	[2,3,16]
Carcinoma en cuirasse	[2,3] CR
Dermatitis	[1,3,4] CR
Hidradenitis suppurativa	[17]
Unique locations	[1-4,13] CR
Eyelids	[3,4]
Inframammary folds	[1,3,4]
Lymphedematous arm	[2]
Radiation therapy port	[2] CR
Scalp	[1]
Scars	[1,2,13]
Subungual	[3]
Umbilicus	[3]
Vascular lesions	[2,3,18-20]
Angiosarcoma	[18]
Carcinoma hemorrhagiectoides	[19]
Carcinoma telangiectoides	[2,3]
Purpura	[3]
Pyogenic granuloma	[20]

Three classic patterns of cutaneous metastases, frequently associated with breast cancer, have been described. They include carcinoma erysipelatoides, carcinoma telangiectoides, and carcinoma en cuirasse. The fourth pattern of metastasis to skin--carcinoma hemorrhagiectoides--has also been observed in a woman with breast cancer [2,3,19].

Carcinoma erysipelatoides also referred to as inflammatory metastatic carcinoma, mimics erysipelas which is an acute streptococcal infection of the skin. Similar to the women in this report, carcinoma erysipelatoides can occur as the sole skin manifestation of cutaneous metastatic breast cancer, or it can appear with other skin patterns of metastatic cancer, such as carcinoma en cuirasse. The cellulitis-mimicking cutaneous metastasis most commonly appears at the prior site of the primary tumor as an erythematous patch and/or plaque with well-defined margins. Pathology shows infiltration of tumor aggregates predominantly in the superficial dermal lymphatic vessels resulting in their obstruction; however, to a lesser degree, the cancer cells can not only be present in the dermal blood vessels but also sparsely infiltrate the dermis. Therefore, the affected skin may also have a peau d’orange (orange peel) appearance--masquerading as an infectious process--resulting from localized lymphedema caused by the blockage of lymphatics by the cancer cell thrombi [2,3].

Carcinoma hemorrhagiectoides is a recently observed pattern of cutaneous metastasis in a breast cancer patient. It typically presents as large, violaceous, confluent, hemorrhagic, and erythematous dermal plaques across the chest from the neck to the abdomen; in some patients, their carcinoma hemorrhagiectoides cutaneous metastases have been described as a shield sign since the presentation of the skin metastasis is reminiscent of a Medieval knight’s shield. In addition, metastatic skin lesions of carcinoma hemorrhagiectoides can also mimic angiokeratomas within the plaques. Pathology shows not only extensive infiltration of the dermis by tumor cells but also endothelial-lined vessels of lymphatic and/or blood origin containing cancer cells. Also, there is hemorrhage of the erythrocytes into the tumor-filled lymph vessels [19].

A 90-year-old woman presented with carcinoma hemorrhagiectoides which appeared as a hot, erythematous, indurated and infiltrated, raised plaque on the left chest wall and breast that extended to her left shoulder, arm, and scapula. Radiologic workup discovered extensive lymph node disease; she also had a markedly elevated lactate dehydrogenase of 1178 international units per liter (normal, up to 460 international units per liter). A skin biopsy demonstrated interconnecting cords and nests of tumor cells in the dermis and subcutaneous tissue; neoplastic cells were also present in the lumina of some of the lymphatic vessels in the superficial dermis. The patient and her family refused any treatment for her metastatic breast cancer; she died six weeks later [19].

Carcinoma telangiectoides appear as red and purple patches and papules usually located on the chest wall; prominent telangiectasias are also typically present. The lesions can mimic those of lymphangioma circumscripta. Pathology shows dilated blood vessels (resulting in the violaceous hue of the lesions) containing tumor cells. The dermis can also be sparsely populated with cancer cells [2,3].

Carcinoma en cuirasse is breast cancer cutaneous metastases that mimic scleroderma. The terminology refers to a cuirassier, who is a cavalry soldier, wearing a cuirass. The cuirass is the fastened-together metal breast and backplate that covers the torso. Cuirass, derived from the French word cuirace (meaning leather), describes the material originally used to make the armor [2,3].

The morphea-like lesions of carcinoma en cuirasse appear as bound-down, flesh-to-red colored scleroderma indurated dermal plaques. Similar to the woman in this report, the lesions can become crusted, ulcerated, or both. Pathology typically shows a linear distribution of single cancer cells between the collagen bundles in the fibrotic dermis [2,3].

In addition to benign-appearing lesions (papules, nodules, and plaques), breast cancer cutaneous metastases can mimic benign cutaneous neoplasms such as cysts, dermatofibromas, or milia-en-plaque [2,5,6]. Cystic skin metastases from breast carcinoma rarely occur. However, a cutaneous metastasis mimicking a cyst was observed on the thigh of a woman with breast cancer [2].

A 68-year-old woman presented with eight-firm brown nodules on her upper back that were suspected to be multiple dermatofibromas; her breast cancer had been treated 26 years earlier. A skin biopsy demonstrated cutaneous metastases of breast origin; her cancer antigen (CA) 15-3 tumor marker was elevated at 426 units per milliliter, but the remainder of her malignancy workup was negative. All the skin lesions disappeared, and her CA 15-3 was negative within 12 months after starting treatment with letrozole [5].

Cutaneous metastases of breast cancer presented as milia-en-plaque on the right breast of a 39-year-old woman whose inflammatory-type infiltrating pleomorphic lobular carcinoma of the left breast with bone metastases had been diagnosed during her pregnancy two years earlier. The cancer was treated with chemotherapy (adriamycin, cyclophosphamide, and docetaxel), surgery (left mastectomy), and radiotherapy. She was receiving trastuzumab when the new skin lesions appeared; in addition to indurated plaques on her sternum and left chest, milia-like papules that resembled mila-en-plaque developed on the undersurface of her right breast. Skin biopsies from the sternum and right breast both showed islands and microthrombi of tumor cells in the dermis; there were no keratin cysts. The subsequent evaluation did not find metastases in the right breast or viscera; the cutaneous lesions were treated with additional radiation therapy, and she was maintained on the trastuzumab [6].

A collision tumor refers to the pathologic presence of two or more benign neoplasms, malignant cancers, or both either juxtaposed or intermingled in the same skin site. Recently, the collision tumor has been renamed as multiple skin neoplasms at one site (MUSK IN A NEST). Two women had breast cancer cutaneous metastases that presented as multiple skin neoplasms at one site: congenital melanocytic nevus and metastatic breast carcinoma [3,4].

A 44-year-old woman presented with a right breast 13 x 8-centimeter fungating tumor of one-year duration that had ulcerated three months earlier; adjacent to the tumor, two non-pigmented nodules (of which the larger was six millimeters) were observed. A right modified radical mastectomy was performed, revealing infiltrating poorly differentiated ductal breast carcinoma involving the nipple skin and all breast quadrants. Metastatic adenocarcinoma was present in the axillary lymph nodes; additional workup revealed liver and bone metastases. Biopsy of the skin nodules showed intradermal nevi; one of the nevi also showed nests of adenocarcinoma cells not only amongst the nevus cells but also in other areas of the dermis and dermal lymphatics [3].

The second patient with multiple skin neoplasms at one site consisting of breast cancer cutaneous metastases and intradermal nevus was a 76-year-old woman with a prior history of breast cancer who presented with a left breast ulcerated mass and a pigmented skin nodule of the upper left chest. The ulcerated breast tumor showed an S-100 negative, cytokine-positive atypical anaplastic tumor consistent with metastatic breast adenocarcinoma. The pigmented skin nodule showed a dermal nevus consisting of S-100 positive, cytokeratin-negative melanocytes in the upper dermis that merged into cords and aggregates of metastatic breast cancer in the deeper dermis [4].

Breast cancer cutaneous metastases can also mimic primary cutaneous malignancies. Breast carcinoma of the inframammary crease can masquerade as a primary basal cell carcinoma or squamous cell carcinoma [4]. In addition, skin metastases from breast cancer can have a similar morphology to keratoacanthoma, melanoma, and pigmented basal cell carcinoma [7,8]. 

A 40-year-old woman was diagnosed with infiltrating ductal carcinoma of the right breast; she had a modified radical mastectomy, and there was no tumor in the axillary nodes. Bone metastases of the left seventh rib were discovered six months later; she received chemotherapy for two years, and they disappeared. They again appeared in the same rib six months after stopping therapy, and she was treated with radiotherapy. Subsequently, three and a half years later, a 7-millimeter brown nodule with irregular borders appeared on the left side of her forehead; the clinical differential diagnosis was melanoma and pigmented basal cell carcinoma. An excisional biopsy was performed. Cutaneous metastatic carcinoma of the breast was demonstrated; the cytoplasm of many of the tumor cells contained large amounts of melanin. Additional patients have also been described with pigmented breast cancer cutaneous metastases on their mastectomy scar or scalp that mimicked a melanoma [7].

Cutaneous breast cancer metastases can mimic bacterial and viral infections. In addition to carcinoma erysipelatoides, bacterial infections mimicked by breast cancer skin metastases include folliculitis. Varicella-zoster virus infection can also be mimicked, presenting as zosteriform metastases, by breast carcinoma spreading to the skin [1-3,9,10].

Folliculotropic cutaneous breast cancer metastases can mimic folliculitis. They presented as painless, non-pruritic, the diffuse pustular-like eruption of four months duration on an erythematous base of the deltoid region of a 51-year-old woman’s right arm who five years earlier had an adenocarcinoma of the breast excised. The lesions, during the patient’s initial evaluation, were determined to be folliculitis. She was treated with topical and systemic antibiotics. When there was no improvement, a biopsy was performed that established the correct diagnosis of folliculotropic metastatic breast carcinoma [9].

Zosteriform metastases describe breast cancer-related skin lesions that are indurated, flesh-colored to red to violaceous, papular, nodular, or pseudo-vesicular and appear in a dermatomal distribution. Similar to the woman in this report, the zosteriform cutaneous metastases can also be crusted or ulcerated. The most common sites affected are the chest and abdominal wall. Although the lesions may be asymptomatic, they can also be painful and thereby result in the patient being treated for herpes zoster virus infection with antiviral therapy before establishing the correct diagnosis [1,3,10].

The cutaneous metastases occur in previously normal skin for some of the patients. However, in other individuals, a prior herpes zoster infection at the location created an immunocompromised cutaneous district that became more susceptible to the subsequent development of lesions at that site. In contrast to varicella-zoster virus-associated lesions, the zosteriform skin metastases may not only occupy more than two to three adjacent dermatomes but also be bilateral [1,3,10].

Several miscellaneous presentations of breast cancer cutaneous metastases have been observed. Similar to the woman with carcinoma en curiasse and zosteriform metastases in this report, the erosive skin metastases can be localized; however, other women have presented with erosive cutaneous metastatic breast cancer that was extensive [2]. Nodular breast cancer cutaneous metastases can also develop ulcers [1]. 

Rarely, women with breast cancer develop clown nose-like cutaneous metastases. This was initially described as diffuse infiltration of the nasal tip; however, the presentation of clown nose skin metastasis has expanded to include exophytic lesions on the tip of the nose. Subsequently, clown nose-like lesions have been observed not only in metastatic visceral tumors (most commonly lung cancer) but also in genetic syndromes (such as Brook-Spiegler syndrome, Hernandez syndrome, multiple familial trichoepitheliomas, tricho-rhino-phalangeal syndrome type I, and tuberous sclerosis) and primary diseases involving the nasal tip [11].

A 74-year-old woman presented with reddish infiltration of the distal part of her nose of three months duration. Five months earlier, she had the removal of her left breast because of cancer. Two months later, she developed a sharply demarcated red infiltration of the nasal tip; palpation of the affected area was painless but very coarse. Biopsy showed metastatic breast carcinoma in the deep dermis and subcutaneous fat extending to the cartilage. Workup did not reveal visceral metastases, and the solitary nasal tip metastasis was excised; however, six months later, she developed brain metastases and died. The investigators emphasized that the coarse consistency of their patient’s nose might be an important indicator of the underlying neoplastic process [11].

Paget’s disease is a primary intraductal mammary carcinoma. However, metastatic breast cancer can also mimic Paget’s disease. In some of these patients, the lesions are pigmented and mimic melanoma [3]. In addition to the areola and nipple, breast cancer cutaneous metastases at other locations on the breast or body can be pigmented and masquerade as melanoma [7].

Papillomatosis cutis lymphostatica (also referred to as elephantiasis nostras verrucosa) is an uncommon asymptomatic manifestation of either primary (hereditary) lymphedema or secondary (acquired) lymphedema; the latter is associated with damaged lymphatics, such as in patients with diabetes. Albeit uncommon, papillomatosis cutis lymphostatica has been observed following iatrogenic secondary lymphedema. Clinically, hyperkeratotic, verrucous, and papillomatous lesions typically develop on the distal aspect of the lymphedematous extremity [12].

Breast cancer cutaneous metastases masquerading as papillomatosis cutis lymphostatica were the initial presentation of recurrent neoplastic disease. A 60-year-old woman developed multiple painless non-confluent oval red nodules that mimicked papillomatosis cutis lymphostatica on the volar aspect of her lymphedematous left arm and forearm. She had a complete mastectomy (without reconstruction) and axillary lymph node dissection one year earlier as the initial treatment of invasive lobular left breast carcinoma; this was followed by local radiotherapy, adjuvant chemotherapy, and currently letrozole hormonal therapy. A skin biopsy was performed since the cutaneous lesions had increased in diffusion and redness during two months of physiotherapy with bandages and plastic devices. The biopsy showed metastasis of invasive breast carcinoma in the dermis and subcutaneous fat [12].

Targetoid breast cancer cutaneous metastases have also been observed. The 54-year-old woman with skin metastases in her radiation therapy port had a targetoid metastasis of her left breast carcinoma. The macular metastasis on the ipsilateral chest presented as an erythematous ring with a white central area (Figures 5, 6).

Metastatic breast cancer presented with targetoid-appearing cutaneous lesions appearing as annular plaques that had depressed hyperpigmented centers in a 40-year-old woman; six months earlier, she had surgery for right breast carcinoma. Examination demonstrated not only a lump on the right breast under the postoperative scar but also papulonodules and targetoid plaques over the right breast and inframammary regions. Fine needle aspiration cytology of a lymph node was suggestive of metastatic breast carcinoma, and biopsy of a skin lesion showed nests of tumor cells [13].

Erythematous breast cancer cutaneous metastases can also masquerade as reactive erythema. Similar to the 54-year-old woman in this report whose skin lesions of metastatic breast carcinoma were in the radiation therapy port, skin metastases can present as confluent erythematous macules that are annular or have irregular-shaped borders. In addition, they can mimic urticaria or erythema annulare centrifugum [3,14,15].

Breast cancer cutaneous metastases--the initial presentation in a previously cancer-free woman--appeared as urticaria on the upper half of the body of a 75-year-old woman. Three months earlier, she had noted the sudden onset of numerous painless, non-pruritic, firm, red, five-millimeter, dermal plaques that were initially considered to be wheals; however, in contrast to bona fide urticaria, the asymptomatic lesions were stable in size and location. A biopsy of the skin lesions showed tumor cells, suggestive of breast cancer, infiltrating the dermis. Subsequent work-up discovered invasive lobular right breast carcinoma--positive for estrogen receptor, progesterone receptor, and HER2--with metastases to bilateral axillary lymph nodes, liver, and bones [15].

Erythema annulare centrifugum is gyrate erythema that typically presents on the trunk and proximal extremities as annular or arcuate patches with an advancing peripheral border and central clearing; at the trailing edge of the erythema, scaling is often present. Erythema annulare centrifugum can be idiopathic; however, it has been associated with multiple conditions including bullous dermatoses (such as pemphigus vulgaris), foods, endocrine disorders (such as Graves’ disease), infections (such as bacterial, fungal, mycobacterial, parasitic, and viral), and medications (such as antibiotics, antihistamines, and antimalarials). Cancer (such as lymphoproliferative malignancies and solid tumors)-associated variant of this reactive erythema has been observed as a malignancy-related condition in patients with breast carcinoma who develop paraneoplastic erythema annulare centrifugum eruption (PEACE). Although the skin lesion of PEACE does not demonstrate cutaneous metastases of breast cancer in the dermis, the appearance of erythema annulare centrifugum correlates with the discovery or recurrence of breast cancer in these oncology patients [14].

Cutaneous metastases of inflammatory breast cancer mimicking erythema annulare centrifugum were the presentation of locally recurrent breast carcinoma in a 38-year-old woman. She presented with several enlarging, annular, erythematous macules with central clearing on the back for which the clinical diagnoses of erythema annulare centrifugum and subacute cutaneous lupus erythematosus were considered. Five years earlier, she had been diagnosed with metastatic invasive ductal right breast carcinoma (that was negative for estrogen receptor, and progesterone receptor, and HER2-positive) involving the dermal lymphatics and lymph nodes. She was treated with chemotherapy, surgery, and radiotherapy. However, 40 months later, she developed inflammatory carcinoma of the left breast with dermal lymphatic and lymph node metastases; surgery was followed by additional chemotherapy and localized radiotherapy. Her new back lesions appeared after completing treatment. Biopsy of the skin lesions showed invasive ductal carcinoma not only in the superficial dermis but also within the lymphatic vessels. At least five additional patients whose locally recurrent cancer presented with metastasis of inflammatory breast carcinoma mimicking erythema annular centrifugum have also been described [14].

Several skin conditions can be mimicked by breast cancer cutaneous metastases. Carcinoma en cuirasse skin metastases from breast cancer can masquerade as scleroderma. In addition, metastatic breast cancer can mimic alopecia areata, dermatitis, and hidradenitis suppurativa [1-4,16,17].

Scalp hair loss caused by cancer is referred to as alopecia neoplastic; it can be primary (from a neoplasm originating within the scalp, such as a dermatofibrosarcoma protuberans or mycosis fungoides) or secondary (from a tumor that has metastasized to the scalp from a visceral organ, such as the breast). Alopecia-associated with breast cancer cutaneous metastases typically presents as patches of absent hair; however, in some individuals, nodules may also be present in the area of alopecia. In some of the breast cancer patients, the cancer-associated hair loss mimics alopecia areata; in other patients with carcinoma of the breast, the neoplasm-related alopecia appears similar to that observed in either discoid lupus erythematosus, lichen planopilaris, morpheaform basal cell carcinoma, or pseudopelade [2,3,16].

Breast cancer cutaneous metastases can mimic dermatitis. Cutaneous metastases located in the skin fold beneath the breasts can masquerade as eczema [1,3,4]. The 54-year-old woman with pruritic skin metastases located in her radiation therapy port considered her new skin lesions to be dermatitis caused by contact of the affected areas with the airbags that occurred during her automobile accident.

A breast cancer cutaneous metastasis that masqueraded as dermatitis and vasculitis was the initial presentation of unsuspected breast carcinoma in a 66-year-old man. The skin lesion, on his left leg for the prior one year, had been clinically diagnosed as dermatitis and vasculitis; he had been unsuccessfully treated with radix salviae miltiorrhizae (also known as danshen, a popular traditional Chinese medicine), other oral medications (antihistamines, antibacterial, and hydrochlorothiazide) and topical corticosteroids. Cutaneous examination of the affected site demonstrated a large solitary erythematous area of nonpitting edema occupying the left buttock and posterior thigh; biopsy of presumptive dermatitis and vasculitis lesion showed metastatic carcinoma in the lymphatic vessels. Subsequently, a thorough clinical evaluation detected not only a previously undiagnosed two-centimeter left breast subareolar mass, but also a swollen lymph node in both the axilla and the groin; lumpectomy of the left breast showed an intraductal carcinoma [3].

Hidradenitis suppurativa is a chronic inflammatory condition that typically presents in the axilla, groin, buttocks, and body fold such as the inframammary creases and chest; lesions appear as tender red nodules and abscesses with fistulas and sinuses. Cutaneous metastatic right breast adenocarcinoma masquerading as hidradenitis suppurativa was the initial manifestation of undiagnosed cancer; it presented as an enlarging red nodule that did not respond to oral and topical therapy for hidradenitis suppurativa in a 30-year-old woman. The woman had draining sinus tracts and skin nodules of seven months duration affecting her axilla, groin, chest, and breasts; a clinical diagnosis of hidradenitis suppurativa was established, and she was treated daily with oral minocycline (100 milligrams) and topical five percent benzoyl peroxide wash and one percent clindamycin solution. At her three-month follow-up visit, all of the skin lesions had resolved except a right breast nodule that had become larger; a biopsy of the nodule showed breast adenocarcinoma [17].

The ipsilateral chest wall and breast (followed by the contralateral chest wall and breast) are the most frequent sites of cutaneous breast carcinoma metastases [1-3]. However, there are several unique sites at which metastatic skin lesions of breast cancer may develop. These include the areola and nipple (mimicking either dermatitis or Paget’s disease), eyelids (with periorbital edema), inframammary folds (masquerading as dermatitis or nonmelanoma skin cancer), subungual distal digit (mimicking an acute paronychia), and umbilicus (as a sister Mary Joseph nodule) [1,3,4]. Cutaneous breast carcinoma metastases can also appear on the scalp (masquerading as alopecia areata or presenting as nodules or both) and the lymphedematous arm [1,2]. Other sites where skin metastases from breast carcinoma occur are in scars (such as that from the mastectomy) and--similar to the 54-year-old woman in this report--within the radiation therapy port where treatment of the primary neoplasm was received [1,2].

In addition to carcinoma telangiectoides (mimicking lymphangioma circumscripta) and carcinoma hemorrhagiectoides (mimicking angiokeratomas), breast cancer skin lesions can also masquerade as other vascular lesions. This includes purpura mimicking vasculitis. It also includes skin metastases that appear similar in morphology to benign and malignant vascular neoplasms, such as pyogenic granuloma and angiosarcoma [2,3,18-20].

A 67-year-old man presented with an easily bleeding, centrally eroded, reddish nodule adjacent to the mastectomy scar on his right chest of four months duration; dermoscopy suggested that the nodule was a hypervascular lesion and the clinical impression was a pyogenic granuloma. Seven years earlier, he had been diagnosed with T2N0M0 papillary right breast carcinoma that was positive for estrogen receptor and progesterone receptor and negative for HER2. He achieved a complete remission following surgery and five years of tamoxifen. The new nodule was excised; pathology showed tubular-glandular structures and aggregates of papillary breast carcinoma that diffusely infiltrated the dermis and subcutaneous fat [20].

Women with either carcinoma erysipelatoides or carcinoma telangiectoides have developed breast cancer cutaneous metastases that mimic primary cutaneous angiosarcoma. A 64-year-old woman presented with a large, indurated, red-purplish plaque--clinically suggestive of angiosarcoma--on the left side of her face, neck, and upper back of four years duration; nine years previously, she had been diagnosed with invasive ductal and lobular left breast carcinoma with bilateral axillary metastases that were treated--resulting in a complete clinical response--with surgery, radiation therapy, and chemotherapy. Biopsy of her skin lesion showed breast carcinoma cell emboli only occupying the dermal lymphatic vessel lumen, without any extravasation of tumor cells into the dermis, consistent with carcinoma erysipelatoides [18].

A 53-year-old woman presented with painful erythema and accompanying pronounced telangiectasia--clinically suggestive of angiosarcoma--extending from the vertex of her scalp (with erosions and focal alopecia) onto the forehead, nose, and cheeks of one-month duration; eight years previously, she had been diagnosed with ductal right breast carcinoma with metastasis in one of eight axillary lymph nodes and was treated--without subsequent clinical or mammographic recurrence until five months earlier--with wide local excision, radiotherapy, chemotherapy, and five years of tamoxifen. Biopsy of the skin lesion showed breast carcinoma tumor aggregates admixed with thrombus cell emboli plugging ectatic blood vessels throughout the dermis, without any extravasation of tumor cells into the dermis, consistent with carcinoma telangiectoides [18].

## Conclusions

Breast cancer cutaneous metastasis most often occurs in oncology patients as a manifestation of persistent or recurrent neoplastic disease. However, less often, they can be the first indication of extranodal metastases in a person with established cancer or the initial presentation of breast carcinoma in a previously undiagnosed individual. The morphology of metastatic breast cancer skin lesions is variable. The pleomorphic skin lesions can have unique locations, and they can mimic benign cutaneous lesions and tumors, cutaneous malignancies, infections, reactive erythema, skin conditions, vascular lesions, and other miscellaneous conditions. A skin biopsy is necessary to confirm the diagnosis of breast cancer cutaneous metastasis. Therefore, especially in a patient with breast cancer and possibly in a cancer-free individual, biopsy should be considered for the evaluation of a new and/or a treatment-unresponsive cutaneous lesion.
